# Physical Medicine & Rehabilitation Physicians’ Confidence Identifying Opioid Misuse Following CDC 2016 Prescribing Guidelines

**DOI:** 10.51894/001c.164035

**Published:** 2026-07-31

**Authors:** Trey P. Heliin, Angela S. Lee, John M. Popovich Jr.

**Affiliations:** 1 College of Osteopathic Medicine, Michigan State University

## 21

### Introduction

In 2016, the Centers for Disease Control and Prevention (CDC) released clinical guideline recommendations regarding physician opioid prescribing. Since then, prescribing rates have declined but remain three-fold than they were in 1999. Limited research has examined the attitudes and beliefs of Physical Medicine & Rehabilitation (PM&R) physicians and their confidence identifying opioid misuse.

### Objectives

The objective of this study is to investigate the impact of the 2016 CDC opioid prescribing guidelines on PM&R physicians’ perceptions of prescribing opioids for patients with chronic low back pain (CLBP). We hypothesize that physicians who completed residency training in 2016 or after possess higher confidence identifying patients at risk for opioid misuse.

### Methods

PM&R physicians with experience treating patients with CLBP were recruited to participate. Physicians completed the Opioid Therapy Provider Survey (OTS), a ten-question survey assessing provider behaviors and confidence in opioid therapy management. Responses were scored on a 5-point Likert scale (1=Completely Disagree, 3=Neutral, 5=Completely Agree). Physicians were grouped based on residency graduation date, with 2016 being the separating year. Individual questions were analyzed using the Mann-Whitney U test with data presented as median (range) and statistical significance set at p < 0.05.

### Results

This cohort included 36 PM&R physicians with experience treating patients with CLBP, where n=30 were men, n=17 were fellowship-trained, n=17 were DOs, and n=19 were MDs. There was no significant difference in the OTS scores between groups in 9/10 questions (*p* > 0.05). However, responses to the question “I can identify patients at risk for misuse of pain medication” were statistically significant between groups. Physicians graduating before 2016 (n=26) reported a median Likert score of 3 (1-5), whereas those who graduated after 2016 (n=10) reported a score of 4 (3-5) (*p* = 0.006), indicating greater confidence in identifying patients at risk among graduates after 2016.

### Conclusion

PM&R physicians who completed training after the release of the 2016 CDC guidelines reported greater confidence in identifying patients at-risk of opioid misuse. Despite the small sample size, these data suggest that opioid prescribing guideline exposure during residency training supports the intended goal of preparing physicians for opioid risk assessment and safer pain management.

